# Factors Influencing the Productivity of Doctors Working in Hospitals in Erzurum Province, Turkey

**DOI:** 10.7759/cureus.75065

**Published:** 2024-12-03

**Authors:** Ahsen Bayraktar, Yusuf Akan

**Affiliations:** 1 Faculty of Economics and Administrative Sciences, Ataturk University, Erzurum, TUR

**Keywords:** erzurum, labour, physician, productivity, sustainability, work satisfaction

## Abstract

Labour productivity, particularly in high-stress sectors like healthcare, is a crucial area of ​​research due to its impact on human lives. The largest volume of health services is undoubtedly provided by public hospitals. In public hospitals, the percentage of the development of hospitals and doctors, and thus the country, constitutes a significant part of the workforce. The aim of this study is to determine the factors that affect the work productivity of doctors working in one of the coldest cities in Turkey, Erzurum. Descriptive statistics, averaging methods, Chi-square test, binary logistic regression analysis, and correlation analysis were applied to the physicians' responses. A questionnaire was completed by 276 physicians working in three hospitals in the city: a University Hospital, a Government Hospital, and a District Hospital. According to the results, doctors' productivity was found to be statistically related to years of working in the hospital, trust, solidarity, and cooperation with colleagues, finding colleagues productive, the architecture of the hospital and the security provided in the hospital, having information about private hospitals in the city, training, congresses and seminars related to the specialty, while despite the cold weather in Erzurum (p<0.05).

## Introduction

Labour productivity is a measure of the amount of output produced by each worker or group of workers at a given standard or desired level. It is an indicator of a country's competitiveness, standard of living, access to information, healthy life expectancy, and economic growth [1]. It is also vital to a country's healthcare system, but it is not easy to achieve high labour productivity. Many factors influence labour productivity, including age, gender, marital status, education, upbringing and physical ability, benefits provided in addition to the worker's fair wage, occupational hazards, illness, and accidents, level of working hours and need for overtime, size of the organization and quality of working life, seniority, skills, values and attitudes, job satisfaction, organizational commitment, motivation, division of labour, technology and total quality management practices [2]. As can be seen from this, the aim should not be to work hard, but to work with high quality. Indeed, when compared with the Organisation for Economic Co-operation and Development (OECD) countries, Turkey has the highest average weekly working time of over 40 hours and is at the top of the OECD ranking with an average of 50 hours per week for men [3]. However, long-term progress cannot be achieved in terms of income and working hours alone. Therefore, the level of development of countries is considered from a broader perspective. In the Human Development Index (HDI), which will be published in 2024, Turkey ranks 45th out of 193 countries [4].

In order to achieve development and welfare as a result of economic growth, it is imperative to address productivity growth in all its dimensions. Productivity is an issue that should be emphasized and developed more for underdeveloped countries, as it affects national income and, thus, the welfare of people [5].

In the 21st century, the issue of health productivity has become the most important indicator of a country's level of development. This is because labour productivity in the production of any good or service is primarily related to the health of individuals. The result of epidemics, early untreated diseases, and injuries is an inefficient workforce. A country's growth in all sectors is therefore linked to its progress in health. The development of the health sector, which is so important, is undoubtedly linked to the improvement of the working conditions of health workers. The better the working conditions of health workers who receive quality training, the better they serve patients. The contribution of workers who receive good service and overcome health problems in the quickest way to productivity at the country level is considerable. Because of their contribution to national labour productivity, the labour productivity of doctors, who are the most highly educated of all health professionals, is one of the most important issues to examine. Productivity in the health sector, as in other professions, is qualified by following innovations, developing up-to-date approaches, and adapting to each country in order to achieve high quality and high performance in the services provided.

Study objective

The aim of this study is to examine the factors that influence the labour productivity of doctors working in public hospitals. To this end, it aims to identify the impact and extent of demographic, psychosocial, administrative, institutional, and physical factors that may affect the labour productivity of doctors, as well as their information about working conditions in other hospitals where they do not work.

## Materials and methods

Study design

The study was a cross-sectional survey. A face-to-face survey or an online questionnaire sent to doctors via email was used to assess their productivity and request their voluntary participation. The Institutional Review Board (IRB) approval was obtained from the Directorate of Social Sciences Institute of Atatürk University with the approval number 13011507004/13.07.2017.

Setting and sample size

This study was conducted among physicians working in three public hospitals in Erzurum province (Palandöken Hospital, Regional Training and Research Hospital, and Atatürk University Hospital), in eastern Türkiye. A total of 972 people, including 602 doctors at Atatürk University Research Hospital, 313 doctors at Regional Training and Research Hospital and 57 doctors at Palandöken Hospital, constitute the population of the study. The sample size of the study was determined using the following formula, and using this formula, the minimum sample size (n) to represent our study population was calculated to be 276, with a margin of error of 5% at the 5% significance level.

The formula used was n= N * P * Q * Z²/ ((N - 1) * d²) + P * Q * Z², [6], where n = sample population size, N = main population volume (number of doctors in three public hospitals in Erzurum), P = efficiency ratio, Q = inefficiency ratio (1 - P), Z = Z-test value at the level of (1 - α) %, α = significance level, d = margin of error (tolerance).

Eligible participants were identified as physicians who were actively working in the three aforementioned hospitals in Erzurum province. Informed consent was obtained from all participants. Voluntary participating physicians aged between 20 and 65 years and officially registered physicians of the hospitals were recruited and included in our study.

Questionnaire

The questionnaire used in the study was obtained through a literature review and consisted of three different sections, with a total of 35 questions. The first section contains demographic information, the second section contains the factors affecting productivity, and the third section contains the doctor's assessment of their own productivity. At the end of the questionnaire, there is a free text area for doctors to write what they would like to add to the subject.

The options of the questionnaire questions applied face-to-face or electronically were determined as ‘Strongly Disagree - Disagree - Undecided - Agree - Strongly Agree’ on the five-point Likert scale and ‘Yes-No’ on the two-point Likert scale. In the section where doctors are expected to rate their own productivity, the ‘Inefficient-Productive’ binary Likert was used.

The study took labour productivity to be the dependent variable and psycho-social, managerial, institutional and physical factors to be the independent variables. Questions about psychosocial factors include teamwork, questions about colleagues, problems outside work, and the productivity of colleagues. Questions related to organizational factors included formal rewards, wages, field-related training, overtime, and shifts. Questions related to administrative factors included managers’ attitudes and behavior, management-employee communication, supervision, doctors’ decisions, and solutions to problems. Questions related to physical factors: Material-technological equipment, weather conditions of Erzurum, transportation to the hospital, architecture of the hospital, and security of the hospital.

Statistical analysis

Data from the study were analyzed using SPSS software, version 20 (IBM Corp., Armonk, NY). Categorical data were presented as frequencies and percentages, and continuous variables were presented as means and standard deviations. The normal distribution of numerical variables was analyzed using skewness and kurtosis values, and values between -1.5 and 1.5 were considered normal. Statistical significance was set at p<0.05.

Reliability analysis

According to the results of the reliability analysis of 50 samples taken from the survey data, the Cronbach's alpha value of our study was 81.7%. Therefore, the scale provides highly reliable results. Kaiser Mayer Olkin (KMO) Bartlett’s test was used to measure the adequacy of correlation between variables. The KMO test is expected to be greater than 0.50 and the p-value obtained is expected to be less than 0.05. The strength values of the questions were analyzed to show how much each factor influences the total factor. At the same time, commonalities were calculated to measure the quality and strength of the question items in the study. The criterion for this is that the extraction results are greater than 0.4.

Logistic regression analysis

There are three main reasons for using logistic regression analysis instead of regression analysis: The dependent variable is continuous in regression analysis, whereas it is discrete in logistic regression analysis, or the probability of the values that the dependent variable may take is estimated in logistic regression analysis; or to use logistic regression, it is not necessary that the independent variables fulfill the normal distribution condition as in regression analysis [7].

When the dependent variable consists of two categories, it is appropriate to use 'binary logistic regression analysis' to examine the relationship between the independent variable and the dependent variable. This analysis method is subdivided into 'single and multiple binary logistic regression analysis'.

In univariate logistic regression analysis, 'productive' and 'unproductive' are classified as dependent variables. Inefficient people, who are the dependent variable, are coded with a value of 1 and efficient people are coded with a value of 2. These values were then coded as '0' and '1' respectively in the SPSS program. According to Chi-square tests, eight independent variables that were found to be related to the dependent variable were included in the analysis. All independent variables are either binary or multi-categorical. For categorical data, the reference group was taken as the first option, and 'indicator/first' was selected in the SPSS software.

The R² value in linear regression, the McFadden R² value in ordinal logistic regression, and the Cox-Snell and Nagelkerke R² values in binary logistic regression models provide information on the suitability of the model. These values should be between 0.20 and 0.40 for a good model fit. To determine the significance of the model, the omnibus test for model coefficients was used and the analysis was performed using the Enter method.

Correlation analysis

In addition to examining the relationship between the independent variables in the model and the dependent variable, i.e., labour productivity, using the Chi-square test and binary regression analysis methods, the relationship between the psychosocial, institutional, administrative, and physical factors represented by the dependent variables and labour productivity as a group was to be examined. This was done by correlating the relationship between the factor groups to which the questions belonged and labour productivity. The correlation coefficient (r) was interpreted as follows: r<0.2, very weak relationship or no correlation; 0.2-0.4, weak correlation; 0.4-0.6, moderate correlation; 0.6-0.8, high correlation; 0.8>, very high correlation.

## Results

Demographic findings

Of the 276 doctors who responded to the survey, 35.14% (n=97) were female and 64.86% (n=179) were male. 98.19% of the participants had a degree from a state university. Doctors in internal medicine were in first place with 55.80% (n=154). Married doctors were in the majority with 67.03% (n=185). Doctors were mostly between 30 and 35 years old with 37.32% (n=103). Doctors who did not have children were in first place with 133 people and a rate of 48.19%. 64.13% (n=177) of the participants had a working life of 1-10 years. The majority of doctors, 65.94%, had worked in their hospital for between one and five years (Table 1).

**Table 1:** Sociodemographic characteristics of the physicians participating in the study

		Frequency	Percent
Gender	Male	179	64.9
	Female	97	35.1
Graduated university	State University	271	98.2
	Private University	5	1.8
Specialization	General practitioner	8	2.9
	Intrinsic sciences	154	55.8
	Surgical sciences	103	37.3
	Basic sciences	11	4.0
Marital status	Married	185	67.0
	Single	91	33.0
Age	20-30	85	30.8
	31-40	103	37.3
	41-50	79	28.6
	>50	9	3.3
Children number	0	133	48.2
	1	55	19.9
	2	61	22.1
	3	23	8.3
	4	4	1.4
Years as a physician	<1 year	6	2.2
	1-10 years	177	64.1
	11-20 years	67	24.3
	21-30 years	21	7.6
	>30 years	5	1.8
Years working in this hospital	<1 year	52	18.8
	1-5 years	182	65.9
	6-10 years	18	6.5
	11-20 years	19	6.9
	>20 years	5	1.8
Working hours per week	<40 hours	17	6.2
	41-75 hours	194	70.3
	76-115 hours	55	19.9
	>116 hours	10	3.6
Number of Patients per day	0-50 patients	175	63.4
	51-100 patients	94	34.1
	101-150 patients	6	2.2
	>151 patients	1	0.4
Total		276	100.0

Distribution of labour productivity factors of doctors

**Figure 1:**
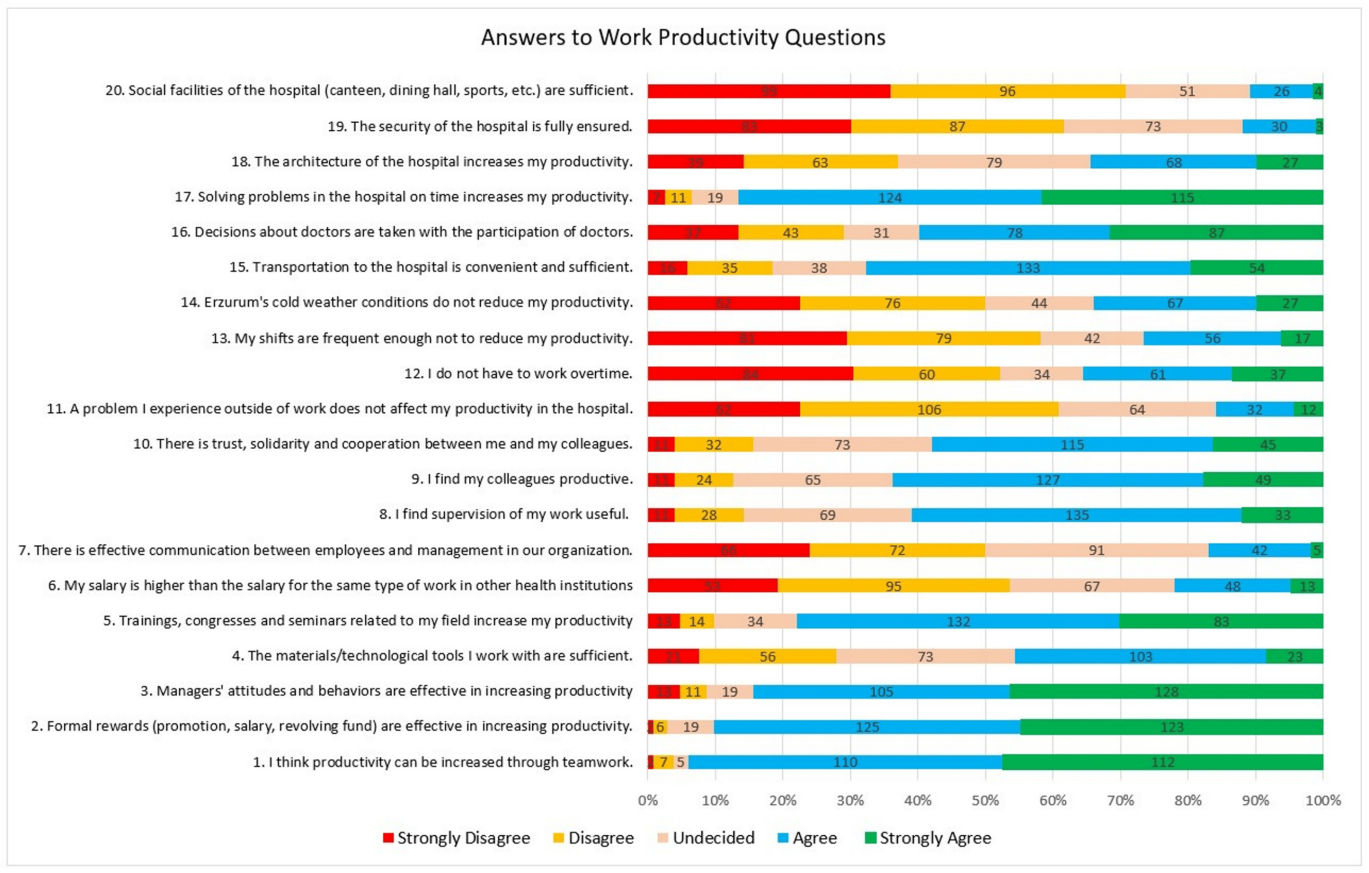
Distribution of physicians' responses to the factors affecting work productivity

The responses of the doctors surveyed to the factors affecting work productivity are shown in Figure 1. For example, 95% of them believe that productivity can be increased through teamwork. Doctors who believe that productivity can be increased through formal rewards represent 89.9% of all doctors. 84.4% of them say that the attitudes and behavior of managers and 77.9% of them say that training, seminars, and congresses related to their specialty increase their productivity. 67.8% of doctors feel that they have convenient and adequate access to the hospitals where they work. Timely resolution of hospital problems increased productivity for 86.6% of doctors. The highest rate of disagreement was the answer that the social facilities of the hospitals in Erzurum are not sufficient with a rate of 70.7%.

Commonalities (strength values of questions) and the means of factors

The calculation of commonalities, which indicates the extent to which each factor influences the total factor and measures the quality and strength of the question items in the survey, showed that the strength ratios exceeded 0.4 for all questions (Table 2). Therefore, the degree to which each of the questions explains the total factor is strong.

**Table 2:** The factor distributions, the strength values of the questions and the means of the responses to the questions

		Extraction	Mean
Psychosocial Factor Questions	1. I think that productivity can be increased through teamwork.	.607	4.46
	9. I find my colleagues productive.	.736	3.65
	10. There is trust, solidarity and co-operation between me and my colleagues.	.738	3.55
	11. A problem I experience outside of work does not affect my productivity in the hospital.	.610	2.37
	20. Social facilities of the hospital (canteen, dining hall, sports etc.) are sufficient.	.738	2.06
Organizational Factor Questions	2. Formal rewards (promotion, wage, revolving fund) are effective in increasing productivity.	.764	4.46
	5. Training, congresses and seminars related to my field increase my productivity.	.710	3.93
	6. My salary is higher than the wage paid for the same type of work in other health institutions.	.565	2.54
	12. I do not have to work overtime.	.580	2.66
	13. My shifts are frequent enough not to reduce my productivity.	.556	2.45
Administrative Factor Questions	3. Managers' attitudes and behaviours increase my productivity.	.515	2.45
	7. There is effective communication between employees and management in our organization.	.673	4.17
	8. I find it useful to be audited about my job.	.537	3.55
	16. Decisions about doctors are taken with the participation of doctors.	.570	3.49
	17. Solving problems in the hospital on time increases my productivity.	.630	4.19
Physical Factor Questions	4. The materials/technological tools I work with are sufficient.	.555	3.18
	14. Erzurum's cold weather conditions do not reduce my productivity.	.612	2.71
	15. Access to the hospital is convenient and sufficient.	.679	3.63
	18. The architecture of the hospital increases my productivity.	.516	2.93
	19. The security of the hospital is fully ensured.	.682	2.21

The Omnibus test for model coefficients was used to determine the significance of the model, and as the analysis was performed using the direct entry method, the step, block, and model Chi-square values were the same. As a result of the analysis, significance levels less than 0.001 indicate that the model is significant (Model Chi-square: 74.153, degree of freedom (Df): 29, p<0.001).

According to the averages obtained, teamwork was found to have the greatest effect on doctors' work productivity among the psychosocial factors. The least influential psychosocial factor is the hospital's social facilities. Among the administrative factors, the timely resolution of problems in the hospital had the greatest effect on productivity, and the attitudes and behavior of managers had the least effect. Among the institutional factors, formal rewards have the greatest effect on productivity; shifts have the least effect on productivity. Among the physical factors, the conditions of transport to the hospital have the greatest effect on productivity; the safety of the hospital has the least effect on productivity (Table 2).

**Figure 2:**
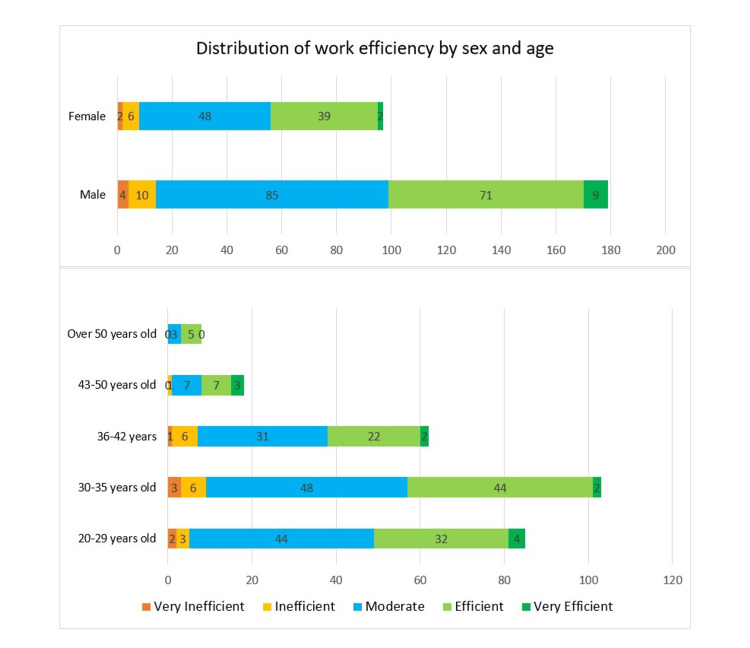
Distribution of feeling productive expressed by the participants according to gender and age groups

The distribution of responses to the question 'To what extent do you feel productive in this hospital?' on the five-point Likert scale by gender is shown in Figure 2. Out of the total, 44.7% (n=80) of 179 male doctors and 42.3% (n=41) of 97 female doctors described themselves as efficient or very efficient. When the productivity results were analyzed by age group, 44 (n=85) of the doctors aged 20-29 years reported that they felt moderately productive. In the 30-35 years age group, which had the largest number of participants (n=103), 48 of the doctors were at a moderate level and 44 of them were at a productive level.

Analysis of factors affecting labour productivity

The relationship between demographic, administrative, psychosocial, institutional, and physical factors affecting doctors' work productivity and their awareness of other hospitals and their work productivity is shown in Table 3. According to the dichotomous Likert question 'efficient/unproductive', 66.5% (n=119) of male doctors, 69.1% (n=67) of female doctors and 67.4% overall felt that they were efficient. According to the statistical analysis, no significant difference was found between the gender of the doctors and their work productivity. Similarly, whether a doctor graduated from a public or private university has no statistically significant effect on productivity. Again, no statistically significant relationship was found between doctors' labour productivity and their specialty, marital status, age groups, number of children, total years of practice, and labour productivity (p>0.05).

**Table 3:** Comparison of productivity status of doctors according to their demographic characteristics * Chi-square test or Fisher's exact test, where available.

		Productivity		Total	P*
		Inefficient (n %)	Efficient (n %)		
Sex	Male	60 (33.5%)	119 (66.5%)	179	0.661
	Female	30 (30.9%)	67 (69.1%)	97	
Graduated university	State university	90 (33.2%)	181 (66.8%)	271	0.116
	Private university	0 (0%)	5 (100%)	5	
Specialization	General practitioner	2 (25%)	6 (75%)	8	0.234
	Intrinsic sciences	56 (36.4%)	98 (63.6%)	154	
	Surgical sciences	31 (30.1%)	72 (69.9%)	103	
	Basic sciences	1 (9.1%)	10 (90.9%)	11	
Marital status	Married	57 (30.8%)	128 (69.2%)	185	0.364
	Single	33 (36.3%)	58 (63.7%)	97	
Age	20-29	22 (25.9%)	63 (74.1%)	85	0.139
	30-35	35 (34%)	68 (66%)	103	
	36-42	27 (44.3%)	34 (55.7%)	61	
	43-50	4 (22.2%)	14 (77.8%)	18	
	>50	2 (22.2%)	7 (77.8%)	9	
Children number	0	43 (32.3%)	90 (67.7%)	133	0.895
	1	18 (47.8%)	37 (48.4%)	55	
	2	21 (34.4%)	40 (65.6%)	61	
	3	6 (26.1%)	17 (73.9%)	23	
	4	2 (50%)	2 (50%)	4	
Years as a physician	<1 year	1 (16.7%)	5 (83.3%)	6	0.334
	1-10 years	59 (33.3%)	118 (66.7%)	177	
	11-20 years	25 (37.3%)	42 (62.7%)	67	
	21-30 years	5 (23.8%)	16 (76.2%)	21	
	>30 years	0 (0%)	5 (100%)	5	
Years working in this hospital	<1 year	6 (11.5%)	46 (88.5%)	52	0.003
	1-5 years	72 (39.6%)	110 (60.4%)	182	
	6-10 years	6 (33.3%)	12 (66.7%)	18	
	11-20 years	4 (21.1%)	15 (78.9%)	19	
	>20 years	2 (40%)	3 (60%)	5	
Working hours per week	<30	7 (41.2%)	10 (58.8%)	17	0.199
	31-55	42 (29%)	103 (71%)	145	
	56-75	16 (32.7%)	33 (67.3%)	49	
	76-95	17 (50%)	17 (50%)	34	
	96-115	7 (33.3%)	14 (66.7%)	21	
	116-135	1 (12.5%)	7 (87.5%)	8	
	>136	0 (0%)	2 (100%)	2	
Number of patients per day	0-50	52 (29.7%)	123 (70.3%)	175	0.284
	51-100	37 (39.4%)	57 (60.6%)	94	
	101-150	1 (16.7%)	5 (83.3%)	6	
	>151	0 (0%)	1 (100%)	1	
Total		90 (32.6%)	186 (67.4%)	276	

Examining the relationship between the total years of service in the hospitals where doctors in Erzurum work and labour productivity, it can be seen that the group with the highest productivity rate is those who have worked for less than one year, and it can be seen that the working hours of doctors in the hospitals where they work are related to their productivity (p=0.003). However, when the relationship between doctors' weekly working hours and work productivity was examined, it was found that there was no relationship between weekly working hours and number of patients per day and work productivity (p>0.05) (Table 3).

Looking at the relationship between productivity and environmental, social, and administrative factors (Table 4), it is clear that there is no relationship between productivity and teamwork, formal rewards (promotion, salary, revolving fund, etc.), attitudes and behavior of managers, adequacy of materials and technological tools, wages received, the effectiveness of communication with management, doctors finding supervision useful, problems with overtime, overtime and number of shifts, attitudes, and behavior of managers, adequacy of materials and technological tools, wages received, the effectiveness of communication between workers and management, doctors' perception of supervision as useful, problems they experience outside work, working overtime, number of shifts, ease of transport to the hospital, participation in decisions taken, timely resolution of problems in the hospital and adequacy of social facilities in the hospital (p>0.05). However, there is a significant difference (p<0.05) between the work productivity of doctors according to the participation of doctors in training, congresses, and seminars related to their own specialty, according to finding their colleagues efficient, finding trust, solidarity, and cooperation with their colleagues, according to the cold weather conditions of Erzurum, according to the architectural structure of the hospital and the security of the hospital.

**Table 4:** Comparison of participants' answers to factors that may affect productivity after being categorized as productive or inefficient * Chi-square test or Fisher's exact test, where available.

		Strongly Disagree		Disagree		Undecided		Agree		Fully Agree		P*
		n	%	n	%	n	%	n	%	n	%	
I think productivity can be increased through teamwork.	Inefficient	1	50	3	42.9	1	20	37	33.6	48	31.6	0.895
	Efficient	1	50	4	57.1	4	80	73	66.4	104	68.4	
Formal rewards are effective in increasing productivity.	Inefficient	2	100	3	50	9	47.4	39	31	37	30.1	0.118
	Efficient	0	0	3	50	10	52.6	87	69	86	69.9	
Managers' attitudes and behaviors increase my productivity.	Inefficient	7	53.8	3	27.3	5	26.3	37	35.2	38	29.7	0.409
	Efficient	6	46.2	8	72.7	14	73.7	68	64.8	90	70.3	
The materials/technological tools I use are sufficient.	Inefficient	11	52.4	16	28.6	27	37	31	30.1	5	21.7	0.176
	Efficient	10	47.6	40	71.4	46	63	72	69.9	18	78.3	
Training, congresses and seminars related to my field increase my productivity.	Inefficient	9	69.2	8	57.1	9	26.5	37	28	27	32.5	0.009
	Efficient	4	30.8	6	42.9	25	73.5	95	72	56	67.5	
The fee is higher than other institutions.	Inefficient	20	37.7	27	28.4	26	38.8	14	29.2	3	23.1	0.500
	Efficient	33	62.3	68	71.6	41	61.2	34	70.8	10	76.9	
There is effective communication between employees and management.	Inefficient	2	100	3	50	9	47.4	39	31	37	30.1	0.118
	Efficient	0	0	3	50	10	52.6	87	69	86	69.9	
I find it useful to have an audit of my work.	Inefficient	5	45.5	7	25	21	30.4	44	32.6	13	39.4	0.662
	Efficient	6	55.5	21	75	48	69.6	91	67.4	20	60.6	
I find my colleagues productive.	Inefficient	8	72.7	8	33.3	26	40	38	29.9	10	20.4	0.009
	Efficient	3	27.3	16	66.7	39	60	89	70.1	39	79.6	
There is trust, solidarity and cooperation between me and my colleagues.	Inefficient	7	63.6	11	34.4	31	42.5	32	27.8	9	20	0.014
	Efficient	4	36.4	21	65.6	42	57.5	83	72.2	36	80	
A problem I experience outside of work does not affect my productivity at the hospital.	Inefficient	23	37.1	32	30.2	24	37.5	8	25	3	25	0.608
	Efficient	39	62.9	74	69.8	40	62.5	24	75	9	75	
I don't have to work overtime.	Inefficient	32	38.1	15	25	14	41.2	20	32.8	9	24.3	0.285
	Efficient	52	61.9	45	75	20	58.8	41	67.2	28	75.7	
My seizures are frequent enough to not reduce my productivity.	Inefficient	30	37	27	34.2	15	35.7	13	23.2	4	23.5	0.421
	Efficient	51	63	52	65.8	27	64.3	43	76.8	13	76.5	
Erzurum's cold weather conditions do not reduce my productivity.	Inefficient	29	46.8	25	32.9	15	34.1	13	19.4	8	29.6	0.025
	Efficient	33	53.2	51	67.1	29	65.9	54	80.6	19	70.4	
Transportation facilities to the hospital are comfortable and sufficient.	Inefficient	8	50	12	34.3	15	39.5	40	30.1	15	27.8	0.403
	Efficient	8	50	23	65.7	23	60.5	93	69.9	39	72.2	
Decisions regarding doctors are made with the participation of doctors.	Inefficient	17	45.9	12	27.9	9	29	28	35.9	24	27.6	0.288
	Efficient	20	54.1	31	72.1	22	71	50	64.1	63	72.4	
Solving problems in the hospital in a timely manner increases my efficiency.	Inefficient	4	57.1	4	36.4	5	26.3	43	34.7	34	29.6	0.548
	Efficient	3	42.9	7	63.6	14	73.7	81	65.3	81	70.4	
The architecture of the hospital increases my efficiency.	Inefficient	20	51.3	23	36.5	25	31.6	17	25	5	18.5	0.028
	Efficient	19	48.7	40	63.5	54	68.4	51	75	22	81.5	
The security of the hospital is fully ensured.	Inefficient	39	47	26	29.9	22	30.1	2	6.7	1	33.3	0.002
	Efficient	44	53	61	70.1	51	69.9	28	93.3	2	66.7	
The social facilities of the hospital are sufficient.	Inefficient	42	42.4	26	27.1	15	29.4	7	26.9	0	0	0.084
	Efficient	57	57.6	70	72.9	36	70.6	19	73.1	4	100	

Whether doctors in Erzurum have information about hospitals other than their own was compared with their work productivity (Table 5). According to this, the majority of the doctors have knowledge about public hospitals other than their own hospital, but the knowledge about other public hospitals in Erzurum or in other cities or private hospitals in other cities does not make a significant difference according to the productivity of the doctors (p>0.05). On the other hand, among doctors working in public hospitals in Erzurum, those who had knowledge about private hospitals in Erzurum had significantly higher productivity (p<0.05).

**Table 5:** Distribution of productivity according to the knowledge of doctors about working conditions of other hospitals where the participants do not work * Chi-square test

			Productivity		Total	P*
			Efficient	Inefficient		
The other state hospitals in this city	Yes	n	54	132	186	0.068
		%	29	71	100	
	No	n	36	54	90	
		%	40	60	100	
Private hospitals in this city	Yes	n	45	120	165	0.027
		%	27.3	72.7	100	
	No	n	44	66	110	
		%	40	60	100	
State hospitals in other cities	Yes	n	54	105	179	0.576
		%	34	66	100	
	No	n	36	81	117	
		%	30.8	69.2	100	
Private hospitals in other cities	Yes	n	42	86	128	0.946
		%	32.8	67.2	100	
	No	n	48	100	148	
		%	32.4	67.6	100	
Total		n	90	186	276	
		%	32.6	67.4	100	

Logistic regression analysis

According to the Chi-square tests, eight independent variables that were found to be related to the dependent variable were included in the regression analysis. In the binary logistic regression model, the Nagelkerke R² value, whose values were used to test the suitability of the model, was found to be 0.330 and it was determined that 33% of the dependent variable could be predicted with this model. The results of the logistic regression analysis carried out after the category coding stage are presented in Table 6. According to the results of the logistic regression analysis, it was concluded that years of work in the hospital (p=0.004), knowledge of private hospitals in the city (p=0.032), and trust, solidarity, and cooperation with colleagues (p=0.045) were significant.

**Table 6:** Results of the logistic regression analysis Nagelkerke R² .330

	F	Std. Error	Wald	Std. Deviation	P	Exp(B)	95% Exp(B)	
							Lower Bound	Upper Bound
Working year			15.369	4	.004			
Working year (1)	2.720	1.231	4.881	1	.027	15.176	1.359	169.441
Working year (2)	.676	1.134	.356	1	.551	1.967	.213	18.152
Working year (3)	.999	1.250	.638	1	.424	2.715	.234	31.474
Working year (4)	1.413	1.298	1.186	1	.276	4.108	.323	52.265
Q 5			5.589	4	.232			
Q 5 (1)	-.967	.783	1.527	1	.217	.380	.082	1.763
Q 5 (2)	-.935	.720	1.685	1	.194	.393	.096	1.611
Q 5 (3)	.434	.544	.635	1	.425	1.543	.531	4.481
Q 5 (4)	.252	.385	.428	1	.513	1.287	.605	2.736
Q 9			2.233	4	.693			
Q 9 (1)	-1.035	.984	1.106	1	.293	.355	.052	2.444
Q 9 (2)	-.606	.747	.658	1	.417	.545	.126	2.359
Q 9 (3)	-.750	.601	1.560	1	.212	.472	.145	1.533
Q 9 (4)	-.673	.538	1.565	1	.211	.510	.178	1.465
Q 10			5.486	4	.241			
Q 10 (1)	-1.099	1.041	1.116	1	.291	.333	.043	2.560
Q 10 (2)	-.351	.682	.264	1	.607	.704	.185	2.683
Q 10 (3)	-1.203	.600	4.015	1	.045	.300	.093	.974
Q 10 (4)	-.589	.564	1.089	1	.297	.555	.184	1.677
Q 14			5.702	4	.223			
Q 14 (1)	-.662	.610	1.176	1	.278	.516	.156	1.707
Q 14 (2)	-.313	.584	.288	1	.592	.731	.233	2.296
Q 14 (3)	-.401	.660	.368	1	.544	.670	.184	2.443
Q 14 (4)	.454	.627	.524	1	.469	1.574	.461	5.374
Q 18			2.685	4	.612			
Q 18 (1)	-1.154	.731	2.495	1	.114	.315	.075	1.320
Q 18 (2)	-.591	.669	.780	1	.377	.554	.149	2.056
Q 18 (3)	-.643	.666	.933	1	.334	.525	.142	1.939
Q 18 (4)	-.488	.677	.519	1	.471	.614	.163	2.314
Q 19			6.676	4	.154			
Q 19 (1)	-.726	1.467	.245	1	.621	.484	.027	8.582
Q 19 (2)	-.132	1.481	.008	1	.929	.876	.048	15.975
Q 19 (3)	.321	1.476	.047	1	.828	.726	.040	13.089
Q 19 (4)	1.365	1.656	.680	1	.410	3.916	.153	100.532
Q 22 (1)	-.699	.327	4.587	1	.032	2.013	1.061	3.817
Constant	1.638	1.919	.728	1	.393	5.146		

If the likelihood ratio of the probability of a doctor being productive is calculated according to the formula (Exp(B)-1)*100, the probability of a doctor working between one to five years is (15.176-1)*100=1417.6. This means that the probability of a doctor working for one to five years is 14% higher than a doctor working for less than one year. A doctor who disagrees that there is trust, solidarity, and cooperation with colleagues is 70% less likely ((0.30-1)*100=-70) than a doctor who agrees. According to formula number 2, the probability that a doctor who has information about private hospitals in the city is more productive than a doctor who does not have information about private hospitals in the city is (2.013-1)*100=101.3. Thus, the probability that a doctor who says yes is 101.3% more likely to be efficient than a doctor who says no.

Correlation analysis

The results of the correlation analyses, which show the relationship between the factor groups to which the questions that represent a factor belong and labour productivity, except for the questions on demographics and knowledge of other hospitals asked of doctors, are presented in Table 7. No statistically significant correlations were found between organizational factors and labour productivity. However, weak positive correlations were found between administrative, physical, and psychosocial factors and labour productivity (r=.135, p=0.025; r=.265 p<0.001; r=.211, p<0.001).

**Table 7:** The correlation matrix

		Workforce Efficiency	Institutional Factors	Administrative Factors	Physical Factors	Psychosocial Factors
Workforce Efficiency	r	1				
	Sig.					
Institutional Factors	r	.039	1			
	Sig.	.522				
Administrative Factors	r	.135	.238	1		
	Sig.	.025	.000			
Physical Factors	r	.265	.192	.403	1	
	Sig.	.000	.001	.000		
Psychosocial Factors	r	.211	.221	.433	.430	1
	Sig.	.000	.000	.000	.000	

Kaiser Mayer Olkin (KMO) Bartlett’s test

The result of the KMO Bartlett's test, which measures the adequacy of the correlation between the variables, was found to be 0.726 and as it was greater than 0.5, it was understood that the data were suitable for factor analysis. In addition, the p-value was found to be <0.001 and it was concluded that the data were normally distributed.

## Discussion

This study examined the factors influencing physicians' work productivity and attempted to identify the effects of demographic, administrative, institutional, and psychosocial factors on productivity. The results showed that doctors' productivity was significantly related to training, congresses, and seminars related to the field, to trust, solidarity, and cooperation among colleagues, to finding colleagues productive, to the architecture of the hospital, and to the security provided in the hospital, while in spite of the cold weather in Erzurum. These results will provide important data for health policy planning and quality improvement, as this study was one of the few studies conducted in Turkey.

There have been some studies in the literature investigating the possible factors influencing physician productivity. In one of the studies on labour productivity and practices in the healthcare sector [8], the author found that increasing competition in the healthcare sector, with a view to controlling costs, is leading to an increase in hospital productivity. Increasing productivity requires the involvement of physicians, administrators, and other staff at all levels. Combining clinical and non-clinical activities and reducing the number of hospital staff are cited as ways of achieving efficiency.

Another study found that while the reforms of the National Health Service in England were intended to provide public funding for health care while improving the efficiency of resource allocation by introducing competition on the supply side of the market, there were some contradictions in the implementation of the reforms [9]. These contradictions have been identified as underutilization of population-based funding, lack of strategy in the development of general practice fund holders who made maverick changes, weak combination of pricing and underwriting rules, capital markets that facilitated cost control but distorted resource allocation; incomplete workforce planning; and multiple market rules.

Frenk et al. examined the problems of excessive unemployment and underemployment in the health sector in urban areas of Mexico through surveys of physicians [10]. The performance of doctors was analyzed based on variables such as social origin and gender, quality of medical education and specialization, and educational production, which was only examined on the basis of demographic variables. In comparison with our study, it is clear that we have assessed the impact of demographic, administrative, institutional, and psychosocial factors on doctors' productivity.

The "Top 500 Most Admired Fortunate" companies studied by Pulde MF understood the concept of "customer comes second" and recognized the relationship between a satisfied workforce and productivity, service quality, and, ultimately, organizational success [11]. If healthcare organizations are to recruit and retain a quality workforce, they must create a strategic plan, organizational structure, and management approach that recognizes that physicians are vital to healthcare.

Research by Bloor and Maynard shows that the National Health Service in the UK has not seriously addressed the issue of productivity for the past 50 years because of its focus on inputs rather than outputs and the lack of information needed to calculate productivity [12]. Today, despite advances in technology, stored information, and changes in organizational structures, productivity has not changed much. The challenge for policymakers is how to improve measures of productivity and how to change the behavior of hospital doctors and general practitioners using the incentives of the National Health Service.

Bunderson JS argues that the psychological contract between employees and the organization is shaped by both professional and administrative work ideologies and thus includes both professional and administrative roles and perceived obligations [13].

One study examined the productivity of doctors working in rural city and county hospitals in China and the factors influencing it [14]. The study found a decline in the average number of inpatients and outpatients per physician, and the reasons for this decline in productivity were evaluated as the decline in the rural population, the recruitment of unsuitable staff, and rapidly increasing health care costs.

In the Netherlands, a study was conducted on the importance of training for health professionals [15]. It examined the extent to which the education received by people working in the public health sector during their academic training was sufficient for their transition to a more complex and variable working life. It was found that the general competencies of new graduates entering public health were better than those in specific fields.

Korkmaz S conducted a study on 40 doctors, 46 nurses, and 34 midwives to measure the motivation of employees who have a great impact on the performance of the hospital and to determine the factors that influence it [16]. In this context, motivational factors were examined under three headings: economic, psychosocial, and organizational-managerial, and it was found that the most important economic motivator for all three groups was money. Among the psychosocial motivators, a rewarding job is effective for doctors and nurses, while security is the most important for midwives. A fair and continuous disciplinary system is important as an organizational and managerial motivator for all three groups. Among the physical motivators, hygiene is the most important motivator for all three groups. While having a laboratory is important for doctors and nurses, having social facilities is more important for midwives.

A study was conducted in Canada to understand the differences in working hours between male and female physicians and the impact of the rapidly increasing number of female physicians on the productivity of all physicians [17]. The average weekly working hours of female physicians was 47.5 hours, compared with 53.8 hours for male physicians. Female doctors were also found to be less likely to be on call, to see fewer patients on call, to take maternity leave, and to be absent more often.

Doğan and Tatlı, conducted a survey on 94 nurses to determine the factors affecting nurses' work productivity [18]. It was concluded that in order to increase the labour productivity of nurses, it is necessary to ensure multidirectional information flow and effective communication between employees and management in hospitals, take necessary measures to reduce the stress of employees, implement an adequate and fair wage system, give more importance to staff training and allocate more resources, reduce the workload of employees and take necessary measures to ensure that employees have timely access to the technology needed in the field, implement management information systems that enable managers to be more effective in decision making, and effectively maintain total quality management practices.

Contarini et al. conducted a study on the causes of demotivation among doctors working in public hospitals in Buenos Aires [19]. A 19-question questionnaire was administered to 155 doctors in seven hospitals in the city. According to the results, work overload, the inability to establish or maintain good communication with colleagues, the belief that their manager is not qualified, and the lack of incentives for learning and research in the hospital were found to be demotivating factors. In this study, 95% of respondents said that salary did not compensate for motivation.

A study by Day et al. surveyed a total of 292 employees in an academic medical center with a pay-for-performance system [20]. The results indicate that rewards distributed according to employee performance have a positive relationship with employee needs. It is demonstrated that employee needs are related to reward distribution in organizational settings other than developing countries or collectivist cultures, and the role of employee-manager communication in the relationship between employee needs and reward distribution.

Stirk and Massoud investigated the importance of improving the performance and productivity of health workers to improve health services. In this study, the factors that influence the performance and productivity of health workers are identified as follows: macro factors such as overall health systems, socio-economic/labour market, and policies; micro factors such as working conditions, communities in which health workers are embedded; Personal characteristics of health workers [21].

In a study by Gaisina et al., it is stated that in order to ensure the effectiveness of the workforce, it is necessary to prevent the deterioration of individual relationships during production [22]. To achieve high productivity, it is necessary to manage human resources and ensure discipline and personal development of employees. The way to achieve this is to ensure that the needs and capabilities of employees are well understood by managers, so that they can express themselves and improve their social status. This will also be important for the sustainability of physician productivity at higher levels.

Another study by Gaisina et al. found that corporate identity has an impact on increasing employee motivation, harmony among employees, work discipline, teamwork, and productivity in an organization. The article identifies the elements on which the formation of the corporate identity of employees depends: Personal ethics, relationships within the team, communication, interaction with the manager, training, motivation, values, traditions, image, and culture of the organization [23].

In all of these studies, the combination of healthcare standards, quality improvement, and input processing regulations was shown to improve the performance and efficiency of healthcare workers. Standards, such as procedures, clinical guidelines, treatment protocols, critical pathways, problem-solving procedures, standard operating procedures, and description of expected health services; quality improvement, such as improving the accountability of workers, making service quality effective, efficient and sustainable, and providing training to ensure competence before and during service; and regulations, such as licensing, standard adjustments, management administration, and performance degradation clauses should be identified. With a particular focus on standards, quality improvement, and regulations to improve the productivity and performance of health workers, this study should examine community involvement, such as provisions for feedback in evaluation, public recognition of performance and contributions, conditions for follow-up by small health committees, and recognition systems, such as individual counselling, performance evaluation, continuing education, reputation, recognition, appreciation, opportunities for career advancement.

Strengths and limitations

This study was conducted in all three major hospitals in Erzurum and achieved the required sample size. In addition to the three different settings, it is one of the few studies in a cold city to examine the effects of weather on physician productivity alongside the effects of demographic, administrative, institutional, and psychosocial factors. However, it has some limitations. Firstly, the study relied on self-reported data, which we cannot validate. Secondly, the results may have been influenced by and reflect the cultural and regional aspects of the study area, which limits the generalizability of the results of the study. Third, the study was not repeated after the changes we found had been adapted and implemented. Further studies are needed to re-evaluate the changes in productivity following the implementation of changes in the key factors identified.

## Conclusions

This research, which was conducted on doctors working in Erzurum State Hospitals, aims to determine the factors that affect the work productivity of doctors. As a result of the evaluations made, it was seen that the model established was appropriate, and it was understood that it was predictable whether a doctor working in Erzurum State Hospitals felt productive or unproductive according to the criteria determined.

Doctors' labour productivity was found to be significantly related to their years of employment in the hospital, the training they received related to their specialty, the productivity of their colleagues, trust and solidarity with their colleagues, the cold weather conditions in Erzurum, the architectural structure of the hospital, the security of the hospital, and having information about private hospitals in the city. When these significant variables were tested with the logistic regression model, it was found that years of working in the hospital, knowledge of private hospitals in the city, and trust, solidarity, and cooperation with colleagues were significantly related to productivity. When the questions are grouped according to the institutional, administrative, physical, and psychosocial factors they represent, and their relationship with work productivity is examined using the correlation method, it can be seen that the physical factors are the most effective and the institutional factors the least effective.

## References

[1] (2024). OECD Compendium of Productivity Indicators.

[2] Doğan M, Tatlı H (2010). Factors affecting labor productivity: an application on nurses in Bingöl state hospital (Article in Turkish). Verimlilik Dergisi.

[3] Liu B, Chen H, Gan X (2019). How much is too much? The influence of work hours on social development: an empirical analysis for OECD countries. Int J Environ Res Public Health.

[4] (2024). United Nations Development Programme (UNDP). Human Development Report 2023-24: Breaking the gridlock: Reimagining cooperation in a polarized world.. New York.

[5] Korkmaz S, Korkmaz O (2017). The relationship between labor productivity and economic growth in OECD Countries. Int J Eco Fin.

[6] Oktay E, Naralan A, Özçomak MS (2010). Investigating the factors affecting the preference of natural gas system in residences: the case of Erzurum (Article in Turkish). Atatürk Üniversitesi Sosyal Bilimler Enstitüsü Dergisi.

[7] Boateng E, Abaye D (2019). A review of the logistic regression model with emphasis on medical research. J Data Anal Info Process.

[8] Griffith JR (1985). Labor productivity in hospitals. Health Matrix.

[9] Maynard A (1994). Can competition enhance efficiency in health care? Lessons from the reform of the U.K. national health service. Soc Sci Med.

[10] Frenk J, Knaul FM, Vázquez-Segovia LA, Nigenda G (1999). Trends in medical employment: persistent imbalances in urban Mexico. Am J Public Health.

[11] Pulde MF (1999). Physician-centered management guidelines. Physician Exec.

[12] Bloor K, Maynard A (2001). Workforce productivity and incentive structures in the UK National Health Service. J Health Serv Res Policy.

[13] Bunderson JS (2001). How work ideologies shape the psychological contracts of professional employees: doctors' responses to perceived breach. J Org Behav.

[14] Martineau T, Gong Y, Tang S (2004). Changing medical doctor productivity and its affecting factors in rural China. Int J Health Plann Manage.

[15] Biesma RA, Pavlova MA, van Merode GA, Groot WB (2007). Using conjoint analysis to estimate employers preferences for key competencies of master level Dutch graduates entering the public health field. Eco Edu Rev.

[16] Korkmaz S (2008). Hastanelerde Doktor Hemşire ve Ebelerin Motivasyonunu Etkileyen Faktörler: Bir Uygulama. [Factors Affecting Motivation of Doctors, Nurses and Midwives in Hospitals: An Application]. Factors affecting motivation of doctors, nurses and midwives in hospitals: an application.

[17] Weizblit N, Noble J, Baerlocher MO (2009). The feminisation of Canadian medicine and its impact upon doctor productivity. Med Educ.

[18] Doğan M, Tatlı H (2010). Factors affecting labour productivity: an application on nurses in Bingöl State Hospital (Article in Turkish). Journal of Productivity.

[19] Contarini PIA, Gilmour AB, González JC, Rebok FD, Cristina VE (2011). Demotivating factors in a sample of public hospital physicians from the city of buenos aires. Prensa Medica Argentina.

[20] Day JWA, Holladay CLA, Johnson SKB, Barron LGC (2014). Organizational rewards: considering employee need in allocation. Personnel Review.

[21] Stirk F, Massoud RM (2015). Improving health worker productivity and performance in the context of universal health coverage: the roles of standards. World Health Organization.

[22] Gaisina LM, Bakhtizin RN, Mikhaylovskaya IM, Khairullina NG, Belonozhko ML (2015). Sociological evaluation of effectiveness of labor workers' behavior. Biosciences Biotechnology Research Asia.

[23] Gaisina LM, Mikhailovskaya IM, Khairullina NG, Pilipenko LM, Shakirova EV (2015). Features of the formation of the corporate identity of the staff. Biosciences Biotechnology Research Asia.

